# Gait characteristics of CKD patients: a systematic review

**DOI:** 10.1186/s12882-019-1270-9

**Published:** 2019-03-06

**Authors:** Damiano D. Zemp, Olivier Giannini, Pierluigi Quadri, Eling D. de Bruin

**Affiliations:** 1 Institute of Human Movement Sciences and Sport, Department of Health Sciences and Technology, ETH Zurich, HCP H 25.1, Leopold-Ruzicka-Weg 4, Zurich, Switzerland; 2 Geriatric Service, Ente Ospedaliero Cantonale (EOC), Regional Hospital of Mendrisio, Via Turconi 23, 6850 Mendrisio, Switzerland; 3 Department of Internal Medicine, Ente Ospedaliero Cantonale (EOC), Regional Hospital of Mendrisio, Mendrisio, Switzerland; 4 Nephrology Service, Ente Ospedaliero Cantonale (EOC), Regional Hospital of Mendrisio, Mendrisio, Switzerland; 5 Division of Physiotherapy, Department of Neurobiology, Care Sciences and Society, Karolinska Institute, Stockholm, Sweden; 6 Department of Internal Medicine and Nephrology Service, Ente Ospedaliero Cantonale (EOC), Regional Hospital of Mendrisio, Via Turconi 23, 6850 Mendrisio, Switzerland

**Keywords:** Chronic Kidney disease, Gait, Mobility, Falls

## Abstract

**Background:**

People with Chronic Kidney Disease (CKD) often present with prevalent gait impairment and high fall rates, particularly in advanced CKD stages. Gait impairment and its consequences is associated with increased hospital admission, institutionalization, and greater need for health care. The objective of this systematic review was to evaluate the quality of studies investigating CKD patients’ gait characteristics at different CKD stages, to highlight areas of agreement and contradiction between studies reporting aspects of gait in CKD, and to discuss and emphasize gait parameters associated with fall risk.

**Methods:**

We performed a literature search of trials in CINAHL (EBSCO), Cochrane Library, EMBASE, Medline (EBSCO), PEDro, PubMed, and Scopus databases from their inception to June 30th 2018 using a two-stage process for the identification of studies. We retrieved English-, German-, Italian-, Spanish-, Portuguese and Dutch-language articles for review. Methodological quality of randomized and non-randomized studies was assessed with an adapted version of the Downs and Black checklist.

**Results:**

Thirty-one studies (22 cross-sectional with 3901 participants) and 9 longitudinal intervention studies (1 randomized control trial, 5 controlled clinical trials and 3 one-group pre-post-test; with 659 participants) were considered. The studies revealed a primary emphasis on gait speed measures within clinical tests, and a neglect of spatiotemporal gait variables. Most of the studies showed that CKD progression is associated with slowing of walking speed. No studies analysed the relation between gait parameters and fall risk.

**Conclusions:**

There was a paucity of studies investigating aspects of gait quality in patients with CKD. In the majority of studies, only gait speed is analysed as a performance indicator. The relation between gait parameters and fall risk in CKD is not investigated. We formulate several recommendations to fill the current research gap, encourage the use of standardized gait analysis protocols that include assessment of spatiotemporal parameters in clinical care of patients with CKD, aimed at prevention of mobility decline and falls risk.

**Electronic supplementary material:**

The online version of this article (10.1186/s12882-019-1270-9) contains supplementary material, which is available to authorized users.

## Background

Chronic Kidney Disease (CKD) is defined as abnormalities in kidney structure or function, present for 3 months, with implications for health [1]. CKD is classified in stages 1 to 5, with higher stages representing lower glomerular filtration rate levels [2]. A potential outcome of CKD is end-stage renal disease (ESRD), requiring costly renal replacement therapy in the form of dialysis or transplantation. CKD ranks 11th place among the leading causes of death globally [3], exhibits unfavourable trends in age-standardized death and disability-adjusted life years (DALYs) rates [4], and its prevalence worldwide is estimated to be 8–16% [5].

Falls in elderly patients on dialysis are common. The falls rate in this population lies between 38 and 47% [6, 7], whereas 1/3 of the senior population not on dialysis fall at least once a year [8]. Recent studies show higher fall rates and fracture risk already in CKD patients not on dialysis [9, 10]. Results from a retrospective cohort analysis seems to indicate, however, that dialysis therapy initiation may be a precipitating factor for falls [11], which might explain in part the higher fall rates seen in this population.

In clinical geriatric practice, instrumental gait analysis [12], often combined with functional tests [13] and instrumental posture analysis [14], is used widely for risk of falls assessment. In this regard, gait assessment considering both physical and cognitive aspects is meaningful because gait requires both kinds of resources [15, 16]. However, conflicting information can be found in the literature about gait in CKD. Where some attribute changes in walking speed to the presence of chronic kidney disease [17], other sources state that these observed changes are due to diseases accompanying some of the CKD patients; e.g. diabetes with peripheral neuropathy [18]. Therefore, a better understanding of gait disorders in CKD patients and the progression of these impairments with increasing CKD severity could be useful to quantify fall risk in this population, and might help identify possible amenable parameters for preventive interventions.

Although there are suggestions that patients show gait abnormalities already in early stages of CKD that lead to heightened risk of falling, little is known about which gait parameters at which stages of CKD could be clinically relevant for fall risk prevention. This systematic review, therefore, focuses on gait characteristics in various stages of CKD and on understanding their possible relation to falls as reported in the scientific literature. We aim in particular to: (1) evaluate the quality of existing studies investigating CKD patients’ gait characteristics at different CKD stages, (2) highlight areas of agreement and contradiction in study results, and (3) analyse the association between gait parameters and falls risk.

## Methods

In March 2017, with an upgrade in July 2018, a professional librarian performed an electronic search of electronic databases from their inception to 2018/06/30 (CINAHL (EBSCO), Cochrane Library, EMBASE, Medline (EBSCO), PEDro, PubMed, Scopus), using a two-stage process for the identification of potential studies.

We combined free-text and Medical Subject Headings terms using a broad range of synonyms, related terms and variant spelling. Second, all reference lists of review articles and included articles were scanned manually. We retrieved English-, German-, Italian-, Spanish-, Portuguese and Dutch-language articles for review.

Three semantic search loops were used (Additional file 1). The first loop contained terms related to kidney disease, the second related to gait and the third included key words that relate to falls. For the identification of relevant falls risk factors, we used two previously published systematic reviews with meta-analyses [19, 20].

### First selection based on abstracts

**Fig. 1:**
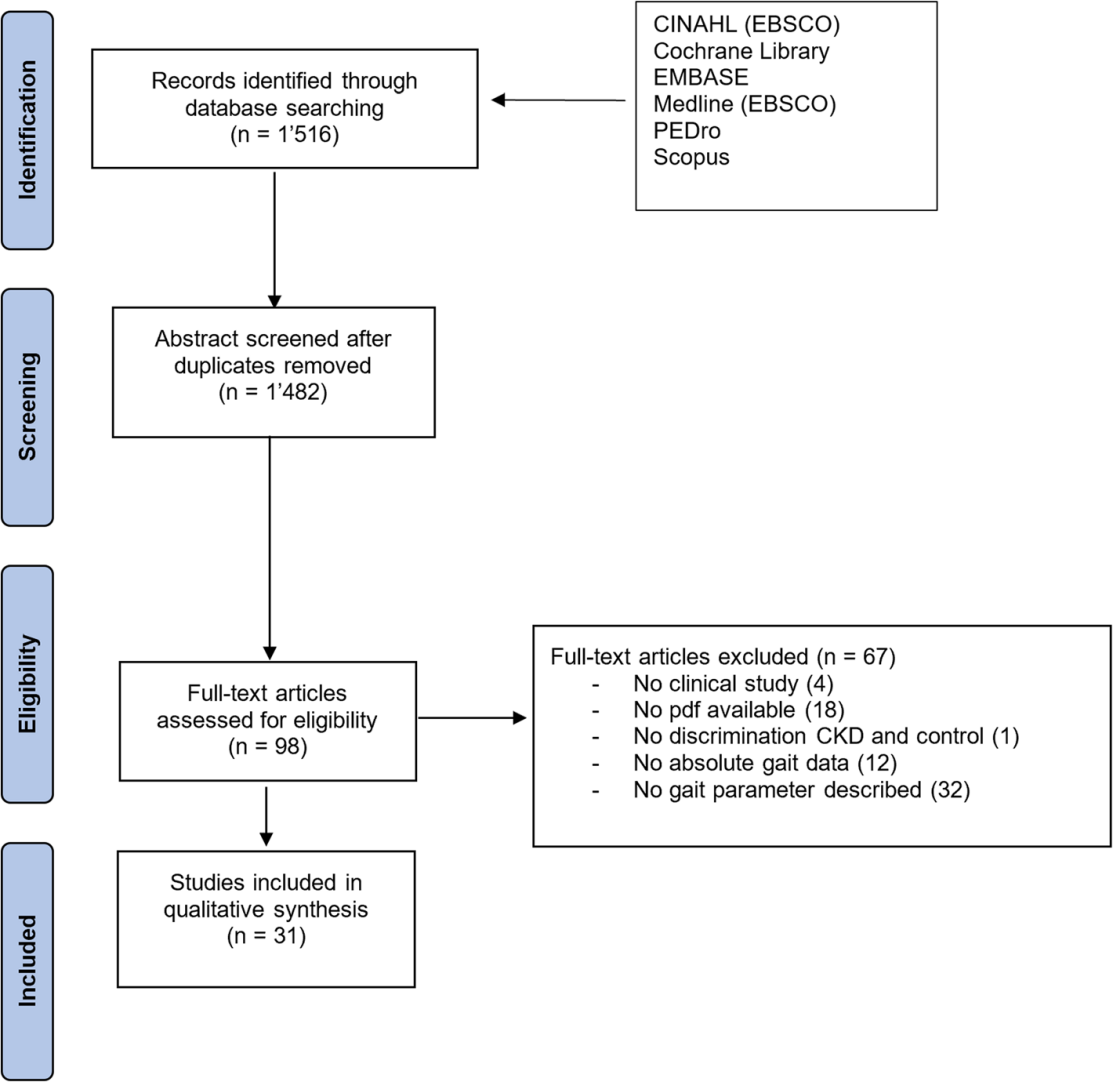
PRISMA flow chart of the articles’ selection process

Two independent reviewers (DZ, EdB) assessed the titles, the abstracts and, where necessary, full texts, to determine the eligibility of each article. Cross-sectional, randomised control trials and controlled clinical trials of human studies were included when (a) a kidney disorder is mentioned; (b) at least one quantitative gait related parameter was measured (e.g. as part of gait analysis or a functional test such as the Short Physical Performance Battery or the Timed Up & Go Test); (c) in English, German, Italian, French, Spanish, Portuguese or Dutch. Studies were excluded when published in an abstract form only. Disagreements between reviewers were resolved by consensus-finding discussion. PRISMA (Preferred Reporting Items for Systematic Reviews and Meta-Analysis) guidelines were followed to ensure the clarity and transparency of reporting of this systematic review [21, 22] (Fig. 1).

### Data extraction

From the eligible manuscripts, the CKD stage, the study population (age, Body Mass Index (BMI), sex), applied selection criteria, gait test, and measured gait parameters, were extracted.

Data were extracted using Microsoft Excel templates purpose-developed for this review. Mean and standard deviation of age, BMI and gait speed in m/s over the different studies were calculated, and the studies were weighted on the basis of the reported number of their participants. Because original individual data were not available no formal statistical comparison to determine whether differences between CKD groups were apparent was possible.

### Quality analysis

Quality of the included articles was assessed by two independent reviewers (DZ, EdB) using Downs and Black checklist for randomized and non-randomized trials [23], which was developed for the assessment of the methodological quality of both randomized and non-randomized studies. We adapted the checklist, discarding redundant items 4, 8, 9 and 17 for cross-sectional studies ([18, 24–44]), and items 14 and 15 for all studies. Item 23 and 24 were considered only for randomized control trials ([45]) and controlled clinical trials ([46–50]) studies. Power (item 27) was considered only in longitudinal intervention studies ([45–53]) and in cross-sectional studies involving a control group ([25, 28, 33, 34, 41–43]). Items 5 and 27 were modified and scored 0 or 1 instead of 0–2 and 0–5 respectively [54]. Adapting the checklist, maximum achievable points differed from one study to another. Therefore, quality score was calculated as the ratio of achieved points to maximum points, indicating 0 for poor and 1 for high quality of the study respectively for each sub-category evaluated.

Kappa statistics was used to calculate inter-rater agreement and interpreted in accordance with Landis and Koch’s benchmarks [55] for assessing the agreement between reviewers: poor (< 0.00), slight (0.00–0.20), fair (0.21–0.40), moderate (0.41–0.60), substantial (0.61–0.80), and almost perfect (0.81–1.00).

## Results

### First selection based on abstracts

Database searching resulted in 1516 articles. After removing duplicates and screening of titles, abstracts and full texts following inclusion criteria, 31 articles with 4560 patients were selected for the review. A summary is given in Fig. 1 and Table 1.

**Table 1:** Overview of articles includes

Article	Population				Selection criteria	Groups matched for	Gait Test	
	Group	n (%men)	Age [years]	BMI [kg/cm^2^]			Distance Start	Speed
Abe 2016 [24]	HD (103.2 ± 103.2)	122 (52.5)	68.0 ± 9.0	21.0 ± 3.7	> 3 months HD	NA	10 m FS*	Max
Blake 2004 [46]	CG HD + PD ([4–25])	12 (58.3) 7 + 5 (58.3)	42.0 ± 9.7 42.0 ± 8.5	24.8 ± 2.5 25.8 ± 3.7	High functioning > 3 months HD	Age, Sex	10 m SND	Norm Max
Bohannon 1994 [25]	CG HD + PD ([0–90]) + CKD	26 (ND) 7 + 17 + 2 (73.1)	44.5 ± 10.3 44.0 ± 9.8	171.0 ± 8.5 cm / 78.8 ± 15.0 kg 171.6 ± 9.5 cm / 80.7 ± 17.0 kg	Transplant candidate	Age, sex, size	20 ft. FS*	Norm
Bohannon 1995 [26]	HD (16.3) + PD (12.3) + CKD	53 + 38 + 20 (69.1)	45.1 ± 11.6	171.9 ± 9.5 cm / 77.6 ± 18.0 kg	Transplant candidate	NA	20 ft. FS	Norm
Bohannon 1997 [51]	CKD	21 (57.1)	42 [21–58]	ND	Transplant candidate	NA	20 ft. FS*	Norm Max
Broers 2015 [27]	HD + PD (ND)	35 + 12 (63.8)	64.8 ± 16.5	M (28.1 ± 4.2) W (25.2 ± 5.6)	> 1 months HD/PD	NA	4 m SS	Norm
Broers 2017 [28]	CG CKD 5 HD / PD (43.2 ± 38.4) HD / PD (6.0 ± 0.0)	20 (65.0) 44 (75.0) 21 + 8 (69.0) 44 (75.0)	59.7 ± 14.1 61.3 ± 12.0 58.2 ± 14.7 ND	24.9 ± 3.4 26.0 ± 4.1 28.1 ± 4.4 25.9 [22.8–28.4]	> 12 months 6 months HD/PD	ND	4 m SS	Norm
Cappy 1999 [52]	HD (58.8 ± 67.2)	16 (62.5)	53.9 ± 15.0	ND	None	NA	28 ft. SND	Norm Max
Chang 2017 [47]	HD (68.7 ± 49.5) HD (49.8 ± 41.7)	21 (61.9) 25 (68.0)	54.2 ± 15.2 54.6 ± 12.7	ND ND	None	NA	6 m SS	Norm
Gordon 2012 [29]	CKD 3–4	13 + 13 (92.3)	61.0 ± 13.0	29.9 ± 6.5	None	NA	20 ft. FS	Norm Max
Headley 2002 [53]	HD (41.6 ± 19.0) *Measured twice in 6 weeks*	10 (70.0)	42.8 ± 4.4	30.3 ± 5.2	None	NA	20 ft. FS	Norm Max
Hiraki 2013 [30]	CKD 2 CKD 3 CKD 4 CKD 5	17 (76.5) 55 (76.4) 25 (68.0) 23 (56.5)	62.4 ± 11.3 66.2 ± 9.9 69.0 ± 9.4 67.6 ± 11.6	24.3 ± 3.2 23.8 ± 3.1 23.1 ± 4.1 22.6 ± 3.0	None	ND	10 m FS*	Max
Jeong 2015 [31]	HD with LVDD HD without LVDD Tot (42.9 ± 37.9)	17 (42.5) 31 (73.8) 48 (58.5)	53.7 ± 12.4 55.2 ± 11.8 54.5 ± 12.0	33.5 ± 7.6 29.5 ± 6.3 31.5 ± 7.2	> 3 months HD	ND	10 m SS	Norm
Jin 2017 [18]	HD diabetic (54.3 ± 49.6) HD no diabetic (81.6 ± 79.1)	35 (68.6) 25 (40.0)	63.9 ± 9.6 56.4 ± 14.7	23.6 ± 3.6 23.2 ± 4.4	None	ND	6 m SS	Norm
Johansen 2001 [33]	CG HD (39.0 ± 39.0)	80 (ND) 39 (66.7)	ND 52.0 ± 16.0	ND 25.5 ± 4.8	None	ND	50 ft. SND	Norm
Johansen 2001 [32]	HD (16.0 ± 16.0)	46 (67.4)	52.0 ± 17.0	25.5 ± 4.6	None	NA	50 ft. SND	Norm
Johansen 2003 [34]	CG HD (34.8 ± 25.2)	19 (63.2) 38 (52.6)	55.0 ± 15.0 55 ± 13.0	24.1 ± 3.6 25.0 ± 4.9	> 3 months HD	ND	20 ft. SND	Norm
Kittiskulnam 2017 [35, 36]	HD (33.6 [15.6–64.8])	645 (58.6)	56.7 ± 14.5	28.1 ± 6.9	> 3 months HD	NA	15 ft. SND	Norm
Kutsuna 2010 [37]	HD (93.6 ± 80.4)	153 (42.5)	64.0 ± 11.0	21.4 ± 3.3	None	NA	10 m FS*	Norm Max
Otobe 2017 [38]	CKD 3a-5 without MCI CKD 3a-5 with MCI	45 (73.3) 75 (78.7)	75.0 ± 5.8 78.7 ± 6.9	23.8 ± 3.5 23.1 ± 4.0	> 65 years old	ND	4 m SND	Norm
Padilla 2008 [39]	CKD 1–5	1 + 3 + 13 + 13 + 2 (84.4)	57.1 ± 14.8	28.1 ± 5.0	None	NA	20 ft. SS	Norm Max
Painter 2000 [48]	HD (33.7 ± 35.6) intervention HD (40.2 ± 62.4) no intervention	ND ND	55.9 ± 15.9 52.8 ± 16.8	ND ND	None	ND	20 ft. SS	Norm Max
Roshanravan 2013 [41]	CG CKD 2–4	78 (60.0) 385 (83.6)	65.4 ± 10.9 61.0 ± 13.0	27.9 ± 5.6 31.0 ± 6.9	None	ND	4 m SS	Norm
Roshanravan 2015 [40]	CKD 1 CKD 2 CKD 3a CKD 3b-5	214 (54.0) 428 (45.0) 138 (31.0) 45 (33.0)	72.0 ± 5.0 73.0 ± 6.0 77.0 ± 7.0 79.0 ± 8.0	27.4 ± 3.8 27.7 ± 4.1 27.0 ± 4.0 27.4 ± 4.8	None	ND	7 m SS	Norm
Rossi 2014 [45]	CKD 3 + 4 CKD 3 + 4	35 (74.5) + 12 (25.5) 42 (72.4) + 16 (27.6)	69.2 ± 12.4 67.7 ± 12.4	30.7 ± 8.7 32.2 ± 7.3	None	ND	20 ft. SS	Norm
Shin 2013 [42]	CG HD (51.6 ± 36.5)	14 (64.3) 14 (71.4)	48.5 ± 10.1 50.0 ± 11.8	29.1 ± 5.8 34.2 ± 8.2	None	Age	7.9 m FS	Norm
Shin 2014 [43]	CG HD diabetic (28.4 ± 21.2) HD no diabetic (70.8 ± 35.6)	18 (72.2) 10 (72.2) 8 (80.0)	51.9 ± 11.0 51.2 ± 10.2 55.5 ± 10.9	29.0 ± 5.3 33.3 ± 6.6 33.0 ± 9.5	None	Age, sex, BMI	7.9 m FS	Norm
Storer 2005 [49]	CG HD HD	12 (58.3) 12 (58.3) 12 (66.7)	44.0 ± 12.0 44.0 ± 9.0 39.0 ± 9.0	25.0 ± 2.0 26.0 ± 3.0 25.0 ± 5.0	None	Age, sex, race	20 ft. FS	Max
Tao 2015 [50]	HD (83.5 ± 61.4) HD (84.7 ± 70.6)	57 (50.9) 56 (53.6)	53.0 ± 11.6 56.7 ± 9.7	ND ND	> 3 months HD	–	10 m SS	Norm Max
Wolfgram 2016 [44]	HD (46.8 ± 48)	28 (71.0)	64.0 ± 10.5	29.3 ± 7.0	> 50 years	–	4 m FS	Norm

*CG* control group, *CKD 1–5* Stage of CKD 1–5, *HD* haemodialysis, *PD* peritoneal dialysis, *SS* standing start, *FS* flying start, FS* supposed flying start, *SND* start not defined, *Norm* self-selected speed, *Max* maximal speed, *LVDD* left ventricular diastolic dysfunction, *ND* not described, *NA* not applicable, *MCI* mild cognitive impairment. For HD and PD months on dialysis are reported. Data are reported as mean ± standard deviation

### Data quality

The agreement on data quality between the two reviewers was high. The estimated Kappa value was 0.81 ± 0.02. The 95% confidence interval ranged from 0.77 to 0.86. The quality scores of studies ranged from 0.28 to 1.00. The mean quality was 0.50 ± 0.15. The mean score was 0.76 ± 0.16 for reporting, 0.19 ± 0.25 for external validity, 0.58 ± 0.22 for internal validity bias, 0.12 ± 0.24 for internal validity, and 0.28 ± 0 .46 for power. The results of quality assessment are summarized in Additional file 2.

### Gait assessment protocols

In all included articles, the gait test assessment consisted of a timed walk during a defined walking course distance. Two publications reported use of a pressure sensitive walkway system (Gaitrite®) that allows more detailed gait analysis; e.g. spatiotemporal gait parameters and their variability [42, 43].

The walk test distance used ranged between 4 m [27, 28, 38, 41] and 50 ft. (15.2 m) [32, 33]. The most used distances were 20 ft. (6.1 m) in 10 articles [25, 26, 29, 34, 39, 45, 48, 49, 51, 53] and 10 m in 6 articles [24, 30, 31, 37, 46, 50]. Whereas gait distance was reported in meters or in feet, speed was always measured using international units m/s or cm/s.

In 11 studies, people performed a standing start [18, 27, 28, 31, 39–41, 45, 47, 48, 50], so acceleration is therefore included in the speed calculation. In 7 manuscripts, a flying start excluding acceleration was reported [26, 29, 42–44, 49, 53]. Thirteen studies failed to describe clearly the start procedure [24, 25, 30, 32–38, 46, 51, 52]; however, in 5 cases [24, 25, 30, 37, 51] we could assume a flying start was used because the authors made reference to other publications using such protocols. Due to the lack of information in many studies about the starting procedure and no apparently significant speed difference in studies using a flying or a standing start, this criterion was not considered in calculating the mean values of each CKD group. In 28 studies participants were asked to walk at a self-selected speed [18, 25–29, 31–48, 50–53]. In 9 of these studies [29, 37, 39, 46, 48, 50–53], people additionally walked at their maximal speed. In one case people walked at self-selected speed under a single- and a dual-task test condition [42], thus considering cognitive contributions to gait [56]. Three protocols measured people at their maximal speed only [24, 30, 49]. The most measured gait parameter was gait speed. Twenty-four of 27 studies only measured this outcome parameter for gait. One publication measured stride time in addition [46]. The two studies that used the sensitive walkway system, measured, along with gait speed and cadence, also the mean and coefficient of variation of stride length, stride time and width; of swing and stance time and of base-of-support and percent of double-support within the gait cycle [42, 43]. An overview of reported gait assessment protocols of the studies is listed in Table 1.

### Population

The mean age of 4560 participants was 61.6 years [42–79], with 23 groups in average older and 37 younger than 60 years. 61.5% were male [31–92%] and the mean BMI was 27.3 kg/m^2^ [21–34]. In 31 publications, we found clinical gait assessment performed in 51 groups of CKD patients and in 9 control groups with 279 participants. Eight groups consisted of patients at a specific CKD stage (one for stage 1 with 214 patients, two each for stages 2 (445 patients) and 3 (193 patients), one for stage 4 (25 patients), and two for stage 5 not on dialysis with 67 patients). The most analysed population was dialysis patients (28 groups with 1673 patients). Fifteen groups with 760 patients where heterogeneous and included patients at different CKD stages.

One publication reported on high functioning HD patients (low comorbidity and self-perceived well-functioning) [46], two compared haemodialysis (HD) patients with and without diabetes [18, 43], one compared HD patients with and without left ventricular diastolic dysfunction (LVDD) [31], two CKD patients with and without diabetes [25, 26] and one CKD patients with and without Mild Cognitive Impairment (MCI) [38]. The remaining publications did not mention exclusion or inclusion criteria, apart from the presence of CKD.

A list of summary descriptions of participants is given in Table 1.

### Gait characteristics

Mean gait speed at self-selected and maximal speed in dialysis patients is lower when compared to control groups and to CKD patients not on dialysis. Mean self-selected gait speed in dialysis groups was between 0.71 and 1.70, with a mean gait speed of 1.12 m/s compared to 1.41 m/s [1.31–1.80] in controls. Maximal gait speed ranged in dialysis groups between 1.47 and 1.86 m/s (mean 1.57), whereas control groups walked maximal at 2.16 m/s [1.89–2.29]. CKD patients not on dialysis have slower self-selected gait speed than controls, which decreases with advancing CKD severity (1.25 ± 0.24 m/s in CKD stage 1, 1.19 ± 0.25 m/s in CKD stage 2 and 1.06 ± 0.28 m/s in CKD stage 3). The same is observed for maximal gait speed, but only up from CKD stages 3 (2.10 ± 0.40 m/s in CKD 3, 1.70 ± 0.50 m/s in CKD 4 and 1.70 ± 0.40 m/s in CKD 5). Although studies with patients on dialysis recorded the modality of replacement therapy, no statistical analysis based on dialysis modality was found. Patients that suffer, along with CKD, from diabetes or LVDD showed generally slower gait speed compared to a matched group free of the second disorder. All gait speed data are summarized in Table 2. One study [28] measured in a 4 m walk test, at self-selected speed, a much higher gait speed when compared to all the other publications: 1.8 m/s for controls, 1.4 m/s for CKD stage 5, and 1.5 m/s, for dialysis patients. Another author [44] reported in HD patients, performing a 4 m walk test at self-selected speed, much slower speed (0.76 m/s) compared to all other HD studies. A detailed description of gait parameters of all studies is available in Additional file 3.

**Table 2:** Gait parameters measured in the 51 groups analysed

Population	Gait speed (m/s)			Stride time (s)
	Self-selected	Maximal	Self-selected under dual-task condition	
Control (*n* = 9)	1.41 [1.31–1.80] *n* = 9/9	2.16 [1.89–2.29] *n* = 4/9	1.28 ± 0.22 *n* = 1/9	1.05 [1.00–1.08] *n* = 3/9
CKD 1 (*n* = 1)	1.25 ± 0.24 *n* = 1/1			
CKD 2 (*n* = 2)	1.19 ± 0.25 *n* = 1/2	2.20 ± 0.20 *n* = 1/2		
CKD 3 (*n* = 2)	1.06 ± 0.28 *n* = 1/2	2.10 ± 0.40 *n* = 1/2		
CKD 4 (*n* = 1)		1.70 ± 0.50 *n* = 1/1		
CKD 5^a^ (*n* = 2)	1.40 [1.2–1.8] *n* = 1/2	1.70 ± 0.40 *n* = 1/2		
Dialysis (*n* = 28)	1.12 [0.71–1.70] *n* = 26/28	1.57 [1.47–1.86] *n* = 9/28	0.87 ± 0.25 *n* = 1/28	1.2 [1.04–1.31] *n* = 4/28

Overall mean and [range] in case of > 1 group or ± SD if only 1 group is described. n = number of groups included

^a^not on dialysis

Stride time was found to be higher in HD-patients compared to controls [43, 46]. The same was found for step time both under single- and dual-task conditions [42].

The complete gait assessment made using a pressure sensitive walkway system (Gaitrite®) showed a much more conservative gait pattern of HD patients compared to controls, with slower gait speed, shorter stride length and longer double-support phase. The difference is much higher in diabetic HD-patients compared to non-diabetic individuals. The HD group showed also a higher stride variability and a higher dual-task cost, especially for gait speed and cadence, when compared to controls [42, 43].

### Falls related gait parameters

No studies analysed the relation between gait parameters and fall risk.

### Intervention studies

Eight studies investigated the effect of different interventions on gait in CKD patients (Table 3). Three studies showed that HD patients can improve self-selected and maximal speed through regular physical activity including flexibility, strengthening and cardiovascular training [50, 52, 57]. Strength training [53] in HD patients and cardiovascular rehabilitation alone demonstrated a positive impact on self-selected gait speed both in HD patients [49] and CKD patients stage 3–4 [45]. The same effect was found in HD patients who underwent physical therapy [45], or performed Tai Chi exercise [47]. One study showed an improved maximal gait speed after kidney transplantation in CKD patients [51].

**Table 3:** Intervention studies

Authors	Intervention	Improvement	Population
Bohannon 1997 [51]	Kidney transplantation.	Maximal gait speed.	CKD
Cappy 1999 [52]	Flexibility, strengthening, cardiovascular training.	Self-selected gait speed and strength.	Haemodialysis
Chang 2017 [47]	Tai Chi.	Self-selected gait speed, physical functioning, quality of life.	Haemodialysis
Headley 2002 [53]	Strengthening.	Strength and normal gait speed.	Haemodialysis
Painter 2000 [57]	Flexibility, strengthening, cardiovascular training.	Self-selected and maximal gait speed.	Haemodialysis
Rossi 2014 [45]	Physical therapy or cardiovascular rehabilitation.	Self-selected gait speed, physical capacity, quality of life.	CKD 3–4
Storer 2005 [49]	Cardiovascular training.	Cardiopulmonary fitness, strength, physical function, gait speed.	Haemodialysis
Tao 2015 [50]	Flexibility, strengthening, cardiovascular training.	Physical function, self-perceived health, self-selected gait speed.	Haemodialysis

## Discussion

The quality of the articles investigating gait characteristics in CKD patients, when scored with our adapted checklist [23], was low to moderate. External and internal validity in particular were evaluated with a low score (see Additional file 2).

Regarding gait assessment, our review uncovers a rather narrow view on it in patients with CKD. When we consider consensus guidelines for gait assessment it becomes apparent that a major gap in current clinical and research knowledge lies in the fact that the assessments methodology varies greatly and does not follow standardized procedures [58]. It seems, therefore, justified to question some of the approaches used for gait assessment in people with CKD because many reports fail to specify the assessment protocol in detail, and studies do or do not include acceleration and deceleration phases of walking in their assessment strategies, and only a few research groups investigated spatiotemporal gait characteristics in standardized settings: e.g. under single- and dual-task conditions.

With the exception of two [42, 43], all studies measured gait speed during walking over a defined distance using a stopwatch. This explains the dearth of information about spatiotemporal gait parameters in CKD patients. Most publications measured gait as a marker for physical performance, and not with the aim of determining gait function per se in people with CKD.

Self-selected walking speed was used in the majority of protocols applied. Three studies measured speed only at maximal walking speed [24, 30, 49]. Only Shin and colleagues were interested in gait quality rather than in performance [42, 43]. For this purpose they used a pressure sensitive walkway system (Gaitrite®) to “ … investigate whether muscle strength is related to gait … ” [43] and to “… examine the DTC^1^ of walking in persons undergoing HD …” [42]. DTC was not evaluated in CKD patients not on dialysis.

Gait assessment under dual task conditions is of great value in determining the severity of gait disorders [59, 60]; it helps to differentiate between central nervous system pathologies and peripheral pathologies [61], and has a prognostic value in the risk of fall [62]. Surprisingly, these gait characteristics have not been assessed in people with CKD. It is well known that many gait pathologies may stem from changes in different brain structures [63], and persons with CKD experience large burden of comorbidities, including cerebral small vessel disease [64], that are related to changes in gait [65]. Furthermore, this is a population with identified heightened fall risk. Based on the high prevalence of cerebrovascular disease in people with CKD it seems fair to hypothesize that gait impairments in the CKD population may be mediated by small vessel disease, and possibly explain part of the increased fall rates seen in these patients.

We also noticed that gait assessment applied to CKD patients is mostly limited to the dialytic population, largely neglecting pre-dialytic groups, even if there are suggestions that gait abnormalities already exist in early stages of CKD that lead to heightened risk of falls. In this regard only 9 studies [28–30, 38–41, 45, 66] out of 31 identified publications measured gait in CKD patients in the pre-dialytic stage (CKD 1–4).

All the studies that compared CKD patients with age-matched healthy controls, showed a significantly slower gait speed in the patients, both at self-selected [25, 28, 33, 34, 42, 43, 46] and at maximal walking speed [25, 46]. Gait speed in CKD patients is associated with physical [24, 25, 34, 35], cognitive [38], sensory [18] and metabolic [29, 43] capacities. Because all of these factors are influenced by CKD severity, slowing down of walking speed seems a logical consequence as shown in Table 2.

The extensive instrumental gait analysis made on a pressure sensitive walkway confirms the influence of cognitive factors on gait quality in people with CKD [42, 43]. While spatiotemporal gait parameters like gait speed, cadence and stride length are partially influenced by muscle strength, and can be seen as markers of gait performance, this is not the case for variability, and dual-task cost of gait, which also depends on cognitive factors [67, 68] and alteration in brain structures [63, 69]. It seems from these publications that HD patients had worse gait variability and dual-task cost of walking compared to control groups. This observation warrants further research into these aspects of gait in people with CKD in various stages of their disease.

Inconsistent results are found in a study where gait speed of controls, CKD stage 5 and HD patients is much higher [28], as compared to values reported in all other studies identified in this review (Additional file 3). Being this the only publication of this review that measured a CKD stage 5 group, Table 2 shows a gait speed in CKD 5 patients similar to the mean of control subjects. The slowest gait speed was measured in a study where HD patients underwent a test battery before and after a dialysis session [44]. Because the studied populations and the used gait test protocol (4 m walk test) of these two publications were not grossly deviant from protocols used in other studies, it is difficult to explain these inconsistencies without further detailed information about the different systems used to assess gait.

Some studies we considered in the review attribute gait changes to diseases accompanying the CKD patients; e.g. diabetes [18, 43], cardiac disease [31] or cognitive impairment [38]; however, the fact that in these same studies the groups free of these comorbidities also showed to have a slower gait speed in comparison to healthy controls, indicates that CKD in itself affects gait, and needs to be considered as an independent risk factor for gait disturbance. The fact that interventional studies described in the review show a positive effect on gait speed of physical therapy training (strengthening, flexibility and cardiovascular training or Tai Chi), or kidney transplantation, hints towards gait changes that are at least partly due to behavioural adaptations, and not solely explainable by CKD.

Gait parameters related to falls are gait speed assessed at self-selected and maximal walking speed [70, 71], stride to stride variability [72–74], and dual-task cost of gait [75–77]. No studies included in this review described a relation between gait performance or gait quality and falls. The higher fall rate of CKD patients on and not on dialysis [6, 7, 9, 10], however, and the deterioration of gait speed performance and quality as described in this review, can be associated with a higher fall risk for CKD patients.

Based on our systematic review, more research is needed for patients with CKD that determines whether and in which stage of the disease changes in spatiotemporal variables become apparent, and whether such change is related to changes in cognition and fall events. We recommend a greater research emphasis on evaluating gait during all CKD stages, and following them using longitudinal study designs.

Spatiotemporal parameters are more sensitive to walking disturbance in clinical populations at higher risk of falls [43, 78], giving important information about motor control, planning and movement strategies. The underlying neurobiological mechanisms of gait changes in CKD are inadequately understood and require investigation. A tenable theory is that part of the changes observed in gait may stem from changes in the brain; however, evidence for this relation in people with CKD is currently lacking. In the case of physical health and fall prevention outcomes, there is evidence from the general older population that spatiotemporal variables of gait bear important additional information.

Long-term assessment of gait in longitudinal study settings may get facilitated through the advent of new inertial measurement units based technology [79, 80]. Feasible wearable sensor technologies are available and could be employed in future studies in clinical settings and people with CKD. We recommend development and use of a standardized assessment [81] for the validation of such assessment to facilitate data comparability between research groups and clinical CKD populations.

### Future direction for studies investigating gait function in CKD

This systematic review reveals that the field of research into aspects of locomotor functioning in people with CKD is still in its fledgling state. Gait disorders, fall risk and cognitive decline in haemodialsysis patients have been analyzed in patients during the course of their therapy for at least 2 years. However, little is known about the transitional period these individuals experience from pre-dialysis to dialysis. It is, therefore, unclear whether these patients entered therapy with pre-existing deficiencies or whether these developed due to treatment initiation. Future studies should, therefore, select a sample of pre-dialytic individuals and describe the changes of gait and cognitive functioning from 1 year before to 1 year after HD beginning. Whether there is an association between muscle functioning during locomotion and neural drive should also be assessed. In a first step this could be assessed using a cross-sectional study design where people on haemodialysis are compared with healthy controls. Such a study could for example employ “intramuscular coherence” or “EMG-EMG coherence” analysis, which considers the common synchronized oscillatory drive to a pair of sEMG placed over the same muscle [82], and reflects the neural drive from the motor cortex to the muscles [82, 83]. Such an approach could substantiate or refute a role of the cortex in locomotor functioning of people with CKD. Whether the type of maintenance haemodialysis plays a role in gait dysfunction is a further intriguing point that may be focussed on. Patients regard the time-consuming nature of haemodialysis as problematic and often fill this time being sedentary and watching tv [84]. It can be hypothesised that this behaviour worsens the physical [85, 86] and cognitive [87] functioning of these patients.

## Conclusions

Our review uncovers a rather narrow view on gait assessment in patients with CKD, with the main focus on determining and reporting gait speed, which decreases with increasing CKD severity. Although there is growing evidence from the general older population that spatiotemporal parameters such as gait cycle variability are more sensitive in terms of psychomotor changes, this is currently not considered in CKD. Instrumental gait analysis to measure spatiotemporal parameters in CKD should be further developed and employed in longitudinal study settings, because these parameters are more informative for fall risk assessment. Further studies are required to analyse gait quality in CKD patients.

## Additional files

Additional file 1: Search strategy (DOCX 13 kb)

Additional file 2: Results of Downs & Black checklist for quality assessment (DOCX 18 kb)

Additional file 3: Summary of gait parameters (DOCX 27 kb)

## Abbreviations

BMI: Body mass index
CKD: Chronic kidney disease
DTC: Dual-task cost
ESRD: End-stage renal disease
HD: Haemodialysis
LVDD: Left ventricular diastolic disfunction
MCI: Mild cognitive impairment
PRISMA: Preferred reporting items for systematic reviews and meta-analysis

## Footnotes

1 Authors’ note: Dual-task cost
