# Supplementary material for: Gait characteristics of CKD patients: a systematic review
Source: BMC Nephrol. 2019 Mar 6;20:83. doi: 10.1186/s12882-019-1270-9 (PMC6404296; doi:10.1186/s12882-019-1270-9)
Supplement: Supplementary file 1 — Search strategy (DOCX 13 kb) [file 12882_2019_1270_MOESM1_ESM.docx]

**Additional file 1** Search Strategy.

| **Loop 1**  **(Kidney disease)** |  | **Loop 2**  **(Gait)** |  | **Loop 3**  **(Falls)** |
| --- | --- | --- | --- | --- |
| CKD OR kidney OR renal OR hemodialysis OR haemodialysis OR dialysis OR eGFR OR GFR OR nephropathy OR uremia OR nephrologic OR nephrological OR glomerul* OR diuresis OR nephrectomy OR renography OR nephritis OR renovascular OR nephrostomy OR pyel* OR creatinine OR dialysate OR “peritoneal dialysis” OR “chronic kidney disease” OR “chronic kidney failure” | AND | gait OR walk OR deambulation OR locomotion OR march OR ambulation OR walking OR kinematic | AND | exercise OR “exercise therapy” OR “accidental falls” OR “fear of falling” OR “falls efficacy” OR “concerns about falling” OR “fall risk” OR fall-risk OR falls-risk OR fall OR falls OR faller OR fallers OR frail OR frails OR frailty OR “physical function” OR “functional performance” OR fitness OR “postural stability” OR “activities of daily living” OR balance OR “functional limitation” OR impairment OR mobility OR “performance test” OR “physical activity” OR “physical activities” OR “physical health” OR “physical inactivity” OR “physical performance” OR power OR proprioception OR “reaction time” OR “response time” OR sway OR mental OR cognit* OR “executive function” OR “executive functions” OR processing OR attention OR “dual task” OR dual-task OR falling |
