# Supplementary material for: Gait characteristics of CKD patients: a systematic review
Source: BMC Nephrol. 2019 Mar 6;20:83. doi: 10.1186/s12882-019-1270-9 (PMC6404296; doi:10.1186/s12882-019-1270-9)
Supplement: Supplementary file 2 — Results of Downs & Black checklist for quality assessment (DOCX 18 kb) [file 12882_2019_1270_MOESM2_ESM.docx]

**Additional file 2** Results of Downs & Black checklist for quality assessment [23].

|  | **Reporting** | | **External validity** | | **Internal validity bias** | | **Internal validity** | | **Power** | | **Total score** | |
| --- | --- | --- | --- | --- | --- | --- | --- | --- | --- | --- | --- | --- |
|  | EdB | DZ | EdB | DZ | EdB | DZ | EdB | DZ | EdB | DZ | EdB | DZ |
| Abe 2016 [24] | 6/7 | 6/7 | 0/2 | 0/2 | 3/4 | 3/4 | 1/4 | 1/3 | 0/0 | 0/0 | 10/17 | 10/16 |
| Blake 2004 [46] | 6/10 | 6/10 | 1/3 | 1/3 | 1/5 | 2/5 | 0/6 | 0/4 | 0/1 | 0/1 | 8/25 | 9/23 |
| Bohannon 1994 [25] | 6/7 | 6/7 | 1/2 | 1/2 | 0/4 | 2/4 | 0/4 | 0/3 | 0/1 | 0/1 | 7/18 | 9/17 |
| Bohannon 1995 [26] | 6/7 | 7/7 | 1/2 | 1/2 | 0/4 | 2/4 | 0/4 | 0/3 | 0/0 | 0/0 | 7/17 | 10/16 |
| Bohannon 1997 [51] | 5/10 | 5/10 | 0/3 | 1/3 | 2/5 | 4/5 | 0/5 | 1/4 | 0/1 | 0/1 | 7/24 | 11/23 |
| Broers 2015 [27] | 5/7 | 5/7 | 0/2 | 0/2 | 1/4 | 2/4 | 0/4 | 0/3 | 0/0 | 0/0 | 6/17 | 7/16 |
| Broers 2017 [28] | 6/7 | 7/7 | 1/2 | 0/2 | 2/4 | 3/4 | 0/4 | 0/3 | 0/1 | 0/1 | 9/18 | 10/17 |
| Cappy 1999 [52] | 4/10 | 3/10 | 1/3 | 1/3 | 4/5 | 4/5 | 1/5 | 0/4 | 0/1 | 0/1 | 10/24 | 8/23 |
| Chang 2017 [47] | 8/10 | 10/10 | 2/3 | 2/3 | 5/5 | 5/5 | 4/6 | 4/6 | 1/1 | 1/1 | 20/25 | 22/25 |
| Gordon 2012 [29] | 5/7 | 6/7 | 1/2 | 0/2 | 2/4 | 2/4 | 0/4 | 0/3 | 0/0 | 0/0 | 8/17 | 8/16 |
| Headley 2002 [53] | 6/10 | 7/10 | 1/3 | 1/3 | 4/5 | 4/5 | 1/5 | 1/4 | 1/1 | 0/1 | 13/24 | 13/23 |
| Hiraki 2013 [30] | 6/7 | 6/7 | 0/2 | 0/2 | 2/4 | 3/4 | 0/4 | 0/3 | 0/0 | 0/0 | 8/17 | 9/16 |
| Jeong 2015 [31] | 6/7 | 6/7 | 0/2 | 0/2 | 2/4 | 2/4 | 0/4 | 0/3 | 0/0 | 0/0 | 8/17 | 8/16 |
| Jin 2017 [18] | 6/7 | 7/7 | 0/2 | 0/2 | 2/4 | 3/4 | 0/4 | 0/3 | 0/0 | 0/0 | 8/17 | 10/16 |
| Johansen 2001 [33] | 4/7 | 6/7 | 0/2 | 0/2 | 2/4 | 2/4 | 0/4 | 0/3 | 0/0 | 0/0 | 6/17 | 8/16 |
| Johansen 2001 [32] | 6/7 | 6/7 | 0/2 | 0/2 | 2/4 | 2/4 | 0/4 | 0/3 | 0/1 | 0/1 | 8/18 | 8/17 |
| Johansen 2003 [34] | 6/7 | 7/7 | 0/2 | 0/2 | 2/4 | 2/4 | 0/4 | 0/3 | 0/1 | 0/1 | 8/18 | 9/17 |
| Kittiskulnam 2017 [35] | 4/7 | 5/7 | 0/2 | 1/2 | 2/4 | 2/4 | 0/4 | 1/3 | 0/0 | 0/0 | 6/17 | 9/16 |
| Kittiskulnam 2017 [36] | 4/7 | 5/7 | 0/2 | 2/2 | 2/4 | 4/4 | 0/4 | 2/3 | 0/0 | 0/0 | 6/17 | 13/16 |
| Kutsuna 2010 [37] | 5/7 | 6/7 | 0/2 | 0/2 | 2/4 | 2/4 | 0/4 | 0/3 | 0/0 | 0/0 | 7/17 | 8/16 |
| Otobe 2017 [38] | 4/7 | 6/7 | 0/2 | 0/2 | 2/4 | 3/4 | 0/4 | 0/3 | 0/0 | 0/0 | 6/17 | 9/16 |
| Padilla 2008 [39] | 6/7 | 6/7 | 0/2 | 0/2 | 2/4 | 3/4 | 0/4 | 0/3 | 0/0 | 0/0 | 8/17 | 9/16 |
| Painter 2000 [48] | 9/10 | 9/10 | 1/3 | 2/3 | 2/5 | 4/5 | 1/6 | 1/6 | 1/1 | 1/1 | 14/25 | 17/25 |
| Roshanravan 2013 [41] | 5/7 | 5/8 | 0/2 | 0/3 | 2/4 | 2/5 | 0/4 | 0/6 | 0/1 | 0/1 | 7/18 | 7/23 |
| Roshanravan 2015 [40] | 3/7 | 7/8 | 0/2 | 0/3 | 2/4 | 2/5 | 0/4 | 0/6 | 0/0 | 0/0 | 5/17 | 9/22 |
| Rossi 2014 [45] | 8/10 | 8/10 | 2/3 | 1/3 | 3/5 | 5/5 | 4/6 | 3/6 | 1/1 | 1/1 | 18/25 | 18/25 |
| Shin 2013 [42] | 5/7 | 5/7 | 0/2 | 0/2 | 2/4 | 2/4 | 0/4 | 0/3 | 0/1 | 0/1 | 7/18 | 7/17 |
| Shin 2014 [43] | 5/7 | 5/7 | 0/2 | 0/2 | 2/4 | 3/4 | 0/4 | 0/3 | 0/1 | 0/1 | 7/18 | 8/17 |
| Storer 2005 [49] | 5/10 | 5/10 | 1/3 | 1/3 | 1/5 | 4/5 | 0/6 | 0/6 | 0/1 | 0/1 | 7/25 | 10/25 |
| Tao 2015 [50] | 10/10 | 10/10 | 3/3 | 1/3 | 5/5 | 5/5 | 6/6 | 5/6 | 1/1 | 1/1 | 25/25 | 22/25 |
| Wolfgram 2016 [44] | 5/7 | 5/7 | 0/2 | 0/2 | 2/4 | 2/4 | 0/4 | 0/3 | 0/0 | 0/0 | 7/17 | 7/16 |
