# Supplementary material for: Gait characteristics of CKD patients: a systematic review
Source: BMC Nephrol. 2019 Mar 6;20:83. doi: 10.1186/s12882-019-1270-9 (PMC6404296; doi:10.1186/s12882-019-1270-9)
Supplement: Supplementary file 3 — Summary of gait parameters (DOCX 27 kb) [file 12882_2019_1270_MOESM3_ESM.docx]

**Additional file 3:** Summary of gait parameters.

| Group | Age | Start  SS/FS | Speed (m/s) | | | Stride time (s) | Author |
| --- | --- | --- | --- | --- | --- | --- | --- |
|  |  |  | Fast | Self-selected | Dual-task |  |  |
| Controls | *< 60* | *-* | *2.05** | *1.59** |  | *1.17** | *Blake 2004 [46]* |
|  | **< 60** | **FS** | **2.24** | **1.42** |  |  | **Bohannon 1994 *[25]*** |
|  | *< 60* | *-* |  | *1.49* |  |  | *Johansen 2003 [34]* |
|  | *< 60* | *-* |  | *1.39* |  |  | *Johansen 2001 [33]* |
|  | **< 60** | **FS** |  | **1.36** |  | **1.08** | **Shin 2014 [43]** |
|  | **< 60** | **FS** |  | **1.35** | **1.00** | **0.50^†^** | **Shin 2013 [42]** |
|  | **< 60** | **FS** | **2.29** | **1.41** |  |  | **Bohannon 1997° [66]** |
|  | **> 60** | **FS** | **1.89** | **1.31** |  |  | **Bohannon 1997° [66]** |
|  | < 60 | SS |  | 1.80 |  |  | Broers 2017 [28] |
| CKD 1 | > 60 | SS |  | 1.25 |  |  | Roshanravan 2015 [40] |
| CKD 2 | **> 60** | **FS** | **2.20** |  |  |  | **Hiraki 2013 [30]** |
|  | > 60 | SS |  | 1.19 |  |  | Roshanravan 2015 [40] |
| CKD 3 | **> 60** | **FS** | **2.10** |  |  |  | **Hiraki 2013 [30]** |
| CKD 3a | > 60 | SS |  | 1.06 |  |  | Roshanravan 2015 [40] |
| CKD 4 | **> 60** | **FS** | **1.70** |  |  |  | **Hiraki 2013 [30]** |
| CKD 5 | > 60 | SS |  | 1.40 |  |  | Broers 2017 [28] |
|  | **> 60** | **FS** | **1.70** |  |  |  | **Hiraki 2013 [30]** |
| Dialysis  (HD and/or PD) | **< 60** | **FS** | **1.86** | **1.32** |  |  | **Headley 2002 [53]** |
|  | **< 60** | **FS** | **1.83** | **1.21** |  |  | **Headley 2002 [53]** |
|  | **< 60** | **FS** |  | **1.00** | **0.87** | **0.60^†^** | **Shin 2013 [42]** |
|  | **< 60** | **FS** | **1.64** |  |  |  | **Storer 2005 [49]** |
|  | **> 60** | **FS** | **1.52** |  |  |  | **Abe 2016 [24]** |
|  | **> 60** | **FS** | **1.52** | **1.14** |  |  | **Kutsuna 2010 [37]** |
|  | **> 60** | **FS** |  | **0.76^1^** |  |  | **Wolfgram 2016 [44]** |
|  | **> 60** | **FS** |  | **0.71^2^** |  |  | **Wolfgram 2016 [44]** |
|  | *< 60* | *-* |  | *0.90* |  |  | Kittiskulnam 2017 [35, 36] |
|  | *< 60* | *-* | *1.74** | *1.31** |  | *1.04** | Blake 2004 [46] |
|  | *< 60* | *-* | *1.47* | *1.04* |  |  | Cappy 1999 [52] |
|  | < 60 | SS |  | 0.99 |  |  | Chang 2017 [47] |
|  | < 60 | SS |  | 1.02 |  |  | Chang 2017 [47] |
|  | *< 60* | *-* |  | *1.13* |  |  | *Johansen 2001 [32]* |
|  | *< 60* | *-* |  | *1.00* |  |  | *Johansen 2003 [34]* |
|  | *< 60* | *-* |  | *1.15* |  |  | *Joahnsen 2001 [33]* |
|  | < 60 | SS | 1.68 | 1.21 |  |  | Tao 2015 [50] |
|  | < 60 | SS | 1.60 | 1.17 |  |  | Tao 2015 [50] |
|  | < 60 | SS |  | 0.91 |  |  | Painter 2000 [48] |
|  | > 60 | SS |  | 1.32 |  |  | Broers 2015 [27] |
|  | > 60 | SS |  | 1.50 |  |  | Broers 2017 [28] |
|  | < 60 | SS |  | 1.70 |  |  | Broers 2017 [28] |
| HD  with diabetes | **< 60** | **FS** |  | **0.90** |  | **1.31** | **Shin 2014 [43]** |
|  | > 60 | SS |  | 0.87 |  |  | Jin 2017 [18] |
| HD  without diabetes | < 60 | SS |  | 1.00 |  |  | Jin 2017 [18] |
|  | **< 60** | **FS** |  | **1.02** |  | **1.23** | **Shin 2014 [43]** |
| HD with LVDD | < 60 | SS |  | 0.70 |  |  | Jeong 2015 [31] |
| HD without LVDD | < 60 | SS |  | 0.90 |  |  | Jeong 2015 [31] |
| CKD 1-5  (no dialysis) | **< 60** | **FS** | **1.79** | **1.10** |  |  | **Bohannon 1997 [51]** |
|  | < 60 | SS | 1.80 | 1.24 |  |  | Padilla 2008 [39] |
| CKD 1-5 + dialysis | **< 60** | **FS** | **1.61** | **1.05** |  |  | **Bohannon 1994 [25]** |
|  | **< 60** | **FS** | **1.76** | **1.09** |  |  | **Bohannon 1995 [26]** |
| CKD 1-5 + dialysis  with diabetes | **< 60** | **FS** | **1.16** | **0.87** |  |  | **Bohannon 1994 [25]** |
|  | **< 60** | **FS** | **1.52** | **1.00** |  |  | **Bohannon 1995 [26]** |
| CKD 1-5 + dialysis  without diabetes | **< 60** | **FS** | **1.90** | **1.14** |  |  | **Bohannon 1995 [26]** |
|  | **< 60** | **FS** | **1.77** | **1.12** |  |  | **Bohannon 1994 [25]** |
| CKD 2-4 | > 60 | SS |  | 0.90 |  |  | Roshanravan 2013 [41] |
| CKD 3-4 | **> 60** | **FS** | **1.76** | **1.26** |  |  | **Gordon 2012 [29]** |
|  | > 60 | SS |  | 1.03* |  |  | Rossi 2014 [45] |
|  | > 60 | SS |  | 1.30* |  |  | Rossi 2014 [45] |
| CKD 3b-5 | > 60 | SS |  | 0.93 |  |  | Roshanravan 2015 [40] |
| *CKD 3a-5 with MCI* | *> 60* | *-* |  | *1.00* |  |  | *Otobe 2017 [38]* |
| *CKD 3a-5 without MCI* | *> 60* | *-* |  | *1.20* |  |  | *Otobe 2017 [38]* |

**Bolt = Flying start (FS)**, unbolt = Standing start (SS), *italic = Start not described*, shadow = age > 60 years (> 60), white = age < 60, * median value, **^†^** step time, ^1^ pre-dialysis, ^2^ post dialysis. ° Reference Data.
